# Local Biomarkers Involved in the Interplay between Obesity and Breast Cancer

**DOI:** 10.3390/cancers13246286

**Published:** 2021-12-14

**Authors:** Jonas Busk Holm, Ann H. Rosendahl, Signe Borgquist

**Affiliations:** 1 Department of Oncology, Aarhus University Hospital, Aarhus University, Palle Juul-Jensens Boulevard 99, 8200 Aarhus, Denmark; 2 Department of Clinical Sciences Lund, Oncology, Lund University, Skåne University Hospital, Barngatan 4, 221 85 Lund, Sweden; ann.rosendahl@med.lu.se

**Keywords:** breast cancer, obesity, overweight, biomarker, microenvironment, progression, initiation

## Abstract

**Simple Summary:**

Breast cancer is the second most common cancer in women worldwide. The risk of developing breast cancer depends on various mechanisms, such as age, heredity, reproductive factors, physical inactivity, and obesity. Obesity increases the risk of breast cancer and worsens outcomes for breast cancer patients. The rate of obesity is increasing worldwide, stressing the need for awareness of the association between obesity and breast cancer. In this review, we outline the biomarkers—including cellular and soluble factors—in the breast, associated with obesity, that affect the risk of breast cancer and breast cancer prognosis. Through these biomarkers, we aim to better identify patients with obesity with a higher risk of breast cancer and an inferior prognosis.

**Abstract:**

Obesity is associated with an increased risk of breast cancer, which is the most common cancer in women worldwide (excluding non-melanoma skin cancer). Furthermore, breast cancer patients with obesity have an impaired prognosis. Adipose tissue is abundant in the breast. Therefore, breast cancer develops in an adipose-rich environment. During obesity, changes in the local environment in the breast occur which are associated with breast cancer. A shift towards a pro-inflammatory state is seen, resulting in altered levels of cytokines and immune cells. Levels of adipokines, such as leptin, adiponectin, and resistin, are changed. Aromatase activity rises, resulting in higher levels of potent estrogen in the breast. Lastly, remodeling of the extracellular matrix takes place. In this review, we address the current knowledge on the changes in the breast adipose tissue in obesity associated with breast cancer initiation and progression. We aim to identify obesity-associated biomarkers in the breast involved in the interplay between obesity and breast cancer. Hereby, we can improve identification of women with obesity with an increased risk of breast cancer and an impaired prognosis. Studies investigating mammary adipocytes and breast adipose tissue in women with obesity versus women without obesity are, however, sparse and further research is needed.

## 1. Introduction

The incidence of breast cancer has risen steadily over the past four decades and is expected to continue [1]. In women, breast cancer is the most common cause of cancer worldwide (excluding non-melanoma skin cancer), with an estimated 2.3 million new cases each year [2]. Risk factors include unchangeable factors (e.g., female gender, age, and genetic mutations) and modifiable factors, such as alcohol consumptions, reproductive factors, physical inactivity, and obesity [3,4]. Since 1975, the prevalence of obesity (body mass index (BMI) ≥ 30 kg/m^2^) has nearly tripled worldwide and the rates continue to rise [5]. Obesity is not only an established risk factor for developing breast cancer but also linked to a higher risk of breast cancer recurrence and mortality [6,7], highlighting the clinical significance of investigating the association.

Various mechanisms have been proposed by which obesity affects the development of breast cancer, where the majority of studies have focused on the systemic alterations associated with obesity [8,9]. Less is known about the role of the local alterations in the breast microenvironment. Focusing on the local composition of the breast, it consists of two major components: glandular tissue and stromal tissue, the latter including fibrous and adipose tissue [10]. On a cellular level, the glandular tissue consists of the epithelial cells producing and passaging milk (lobules and ducts). In the development of breast cancer, these epithelial cells transform into cancer cells. A plethora of crosstalk between the epithelial cells and the stromal tissue highlights the importance of the local microenvironment in the breast regarding the development of breast cancer [11,12]. In this review, we aim to provide an overview of obesity-associated local biomarkers in the breast associated with breast cancer initiation and progression, including findings from both in vitro and in vivo, and human studies. The major part of the review will focus on breast cancer progression. First, we will briefly address changes in the adipose tissue of the breast associated with obesity. Second, we will outline biomarkers associated with the initiation of breast cancer among women with obesity. Third, biomarkers associated with breast cancer progression (growth, proliferation, migration, invasion, etc.) in the breast in obesity (from now on referred to as “obese breast”) are presented. Finally, future perspectives in the research of local obesity-associated biomarkers and breast cancer are discussed. 

## 2. Obesity-Associated Changes in Breast Tissue

When addressing the effect of local obesity-associated changes on breast cancer, it can be divided into two processes; breast cancer initiation and breast cancer progression (e.g., proliferation, growth, invasion, and migration). So far, the association between the local obese environment and breast cancer progression is the most explored of the two processes. It is still unresolved which mechanisms are involved in the initiation of breast cancer [3].

**Figure 1:**
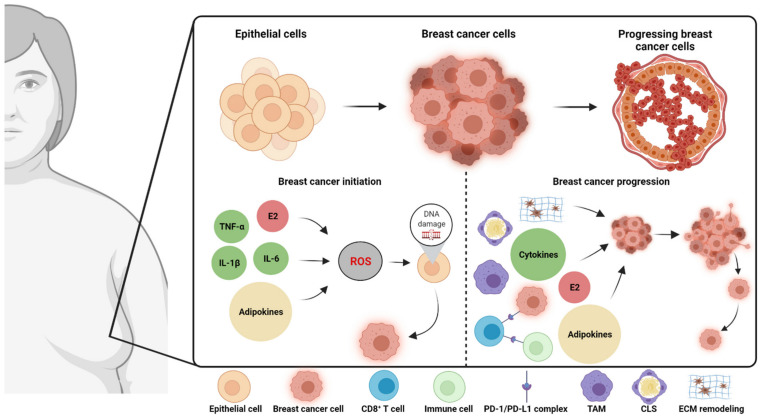
Local obesity-associated biomarkers involved in breast cancer initiation (left panel) and breast cancer progression (growth, proliferation, invasion, migration, etc., right panel). Left panel: In the obese breast, the secretion of pro-inflammatory cytokines is increased. TNF-α, IL-1β, and IL-6 are associated with an increased production of ROS, which could induce DNA damage in the mammary epithelial cell, and, thereby, the initiation of breast cancer. Studies on obesity-associated adipokines (leptin, resistin, and fatty acid-binding protein 4) have shown similar mechanisms. In addition, the level of 17β-estradiol is increased, which induces DNA damage, mainly through ROS production. Right panel: Altered levels of pro-inflammatory cytokines, various adipokines, and 17β-estradiol may lead to tumor progression in the obese breast. Immune cells (macrophages and CD8^+^ T cells) are associated with breast cancer progression, too. Remodeling of the extracellular matrix (involving matrix metalloproteinases and collagens) influences tumor progression, for example, through the promotion of invasion and metastasis. Abbreviations: E2 = 17β-estradiol; IL = interleukin; TNF-α = tumor necrosis factor alpha; TAM = tumor-associated macrophages; ECM = extracellular matrix; CLS = crown-like structures; ROS = reactive oxygen species; PD-L1 = programmed death-ligand 1; PD-1 = programmed cell death 1. Created with BioRender.com by the authors.

The adipose tissue is a major component of the breast, but the percentage of adipose tissue varies widely between individuals, depending on the variation in stromal and glandular tissue [13,14]. Despite interpatient variability, breast cancer develops in an adipose-rich microenvironment, with the adipose tissue being critical for the normal development of the mammary gland [14]. Adipose tissue depots vary in function and composition in different regions of the body [13], but how the mammary adipose tissue differs from other adipose depots is not fully understood [8]. White adipose tissue is roughly divided into visceral and subcutaneous adipose tissue [8]. Despite its unique tissue-specific functions, the adipose tissue in the breast is considered subcutaneous white adipose tissue [13,15]. The cellular composition and changes in white adipose tissue (the mammary adipose tissue) associated with breast cancer initiation and progression in obesity are briefly outlined (Figure 1) and further described in detail in the following sections.

The breast adipose tissue consists of adipocytes (mainly white adipocytes), adipose precursor cells (pre-adipocytes), immune cells, endothelial cells, fibroblasts, pericytes, and extracellular matrix [16]. In normal-weight conditions, the adipose tissue is rich in anti-inflammatory immune cells, such as M2 macrophages and regulatory T cells, and anti-inflammatory cytokines, such as interleukin 10 (IL-10) [17]. In obesity, the adipose tissue expands and changes the homeostasis of the adipose tissue, resulting in adipose tissue dysfunction with deregulated adipokine, cytokine, and growth factor secretion [8,17]. Oxygen demands exceed the supply, leading to hypoxic conditions, adipocyte cell death, and a shift towards a pro-inflammatory state [18,19]. The immune cell landscape changes with the invasion of the pro-inflammatory M1 macrophages and CD8^+^ T cells, among others [17,18]. The secretion of pro-inflammatory adipokines and cytokines, such as leptin, IL-1, IL-6, and tumor necrosis factor alpha (TNF-α), is also increased [8,17]. The pro-inflammatory state contributes to an increase in aromatase activity, hereby inducing elevated levels of estrogen [20]. Obesity is further associated with increased production of reactive oxygen species (ROS), which could contribute to the initiation of breast cancer in the obese breast through cellular DNA damage [21,22,23]. In addition, hypertrophic adipocytes and white adipose tissue inflammation result in extracellular matrix (ECM) remodeling and chronic fibrosis that may foster tumor establishment and progression [18,24,25]. 

In summary, obese adipose breast tissue is associated with a pro-tumorigenic local environment through hypoxic areas, inflammation, altered adipokine secretion, changes in aromatase activity, and ECM remodeling. All of the above-mentioned local obesity-associated biomarkers play a role in breast cancer initiation and progression and will be reviewed in detail in the following sections. 

## 3. Breast Cancer Initiation and Local Obesity-Associated Biomarkers

### 3.1. Initiation of Breast Cancer in Obesity

Mutations in the DNA and genomic instability are key features in the initiation of cancer [26]. In a recent review by Wlodarczyk et al. [27], the authors address the potential link between obesity and genomic instability, leading to mutations that result in tumorigenesis and the formation of cancer cells [27,28]. Mutations in the DNA are known to result from the production of ROS and other types of metabolites that cause DNA damage [22,29,30]. ROS are chemical species formed upon incomplete reduction of oxygen and include free radicals such as superoxide and hydroxyl radical [27,31]. In obesity, ROS production is induced by, for example, increased uptake of glucose and fatty acids, which activates an NADPH oxidase isoform (NOX4) in adipocytes [27]. However, the direct link between the obese environment in the breast and breast cancer initiation is poorly investigated and remains hypothetical [3,32]. Nonetheless, some obesity-associated biomarkers are associated with ROS production and DNA damage and could partly explain the initiation of breast cancer in the obese breast, as addressed by an excellent recent review by Bhardwaj et al. [3]. As the majority of studies reporting obesity-associated biomarkers regarding ROS production are of non-mammary origin, this link remains to be investigated in mammary epithelial cells [3]. Therefore, the studies included address the biomarkers involved in ROS production and DNA damage in various cell types, not only mammary epithelial cells. In the following section, we address obesity-associated biomarkers in the breast with mutagenic potential seen in different cell types.

### 3.2. Local Obesity-Associated Biomarkers and DNA Damage

#### 3.2.1. Inflammation

In the obese breast, a state of chronic low-grade inflammation is commonly found [12,18,33]. During adipose tissue expansion, adipocytes become hypertrophic and eventually die. This releases damage-associated molecular patterns (DAMPs), which leads to a pro-inflammatory state [18]. Infiltration and activation of pro-inflammatory immune cells (such as M1 macrophages, neutrophils, and CD8^+^ T cells) lead to accumulation of cytokines (IL-1β, IL-6, and TNF-α, among others) with the potential to induce DNA damage via the generation of ROS [3,18,34,35,36,37,38,39,40,41,42]. TNF-α increases ROS production, potentially contributing to the initiation of breast cancer [35,36]. Increased ROS levels by TNF-α have been found in myocardial cells and liver cells [37,38]. IL-1β induces a significant ROS production in different studies examining chondrocytes [39,40,41]. According to Ji et al. [42], another interleukin, IL-6, increased the intracellular production of ROS in normal 3T3-L1 adipocytes. In conclusion, inflammation can act mutagenic through a pro-inflammatory cytokine-mediated increase of ROS production, which could lead to DNA damage and potentially initiation of breast cancer.

#### 3.2.2. Estrogen

Aromatase is the rate-limiting enzyme for estrogen biosynthesis and is expressed in the adipose tissue in the breast and other adipose tissue depots [43]. After menopause, adipose tissue becomes the predominant site for conversion of estrogen precursors to 17β-estradiol, the most potent form of estrogen [3,8,44]. Morris et al. [45] found a correlation between elevated BMI and increased expression and activity of aromatase in white adipose breast tissue. In the obese adipose tissue, aromatase activity increases, driven by, for example, inflammatory factors (IL-1β, IL-6, TNF-α, and prostaglandin E2), leptin, and also through an increase in the number of adipose cells [3,46,47,48,49,50]. Hence, the local levels of estrogens in the breast are elevated in women with obesity compared to women without obesity. The role of estrogen in breast cancer initiation has been outlined in two reviews by Bhardwaj et al. [3,48], with key points presented below. 

Estrogens can stimulate DNA damage in different ways [3,51]. First, the metabolism of estrogens forms catechol estrogen metabolites inducing the production of ROS through redox cycling [51]. Second, these catechol estrogen metabolites can interact with the DNA directly and form depurinated sites, resulting in point mutations in the DNA [51]. Third, estrogens can impair the DNA damage response in the cell, for example, by inhibiting key initiators of the response, such as the effector kinases ATM and ATR [52]. In addition, estrogens increase cell proliferation, further challenging the DNA damage response and repair capacity [3,52].

#### 3.2.3. Adipokines (Leptin, Resistin, and Fatty Acid-Binding Protein 4)

Adipokines are soluble factors produced by adipocytes, with more than 600 adipokines identified so far [53]. In the obese state, adipocytes produce an imbalanced amount of adipokines [53]. Below, the role of the adipokines leptin, resistin, and fatty acid-binding protein 4 (FABP4) in ROS production and DNA damage will be reviewed. 

In the adipose tissue, adipocytes are the primary source of leptin production, but pre-adipocytes also produce leptin [3]. In circulation, the levels of leptin are positively associated with BMI [54,55]. In the local environment of the breast, adipose stromal cells (pre-adipocytes) produce leptin as the adipose tissue expands during weight gain [56]. Leptin can initiate breast cancer by enhancing the expression of the above-reviewed mediators and through other mechanisms. By upregulating the expression of IL-1β, IL-6, and TNF-α, leptin can induce ROS production, as described above [25,56]. Further, leptin can increase levels of estrogens in the local environment by stimulating aromatase expression in pre-adipocytes and F442A adipocytes [49,57]. Liu et al. found that injection of leptin in leptin-deficient obese mice and lean mice increased aromatase expression in the adipose tissue [49]. Furthermore, mRNA expression of aromatase in the adipose tissue was significantly lower in leptin-deficient obese mice than in matched wild-type lean mice [49]. Additionally, injection of leptin in both the obese and lean mice increased the level of aromatase expression. Therefore, leptin increases levels of estrogens and induces DNA damage through the various mechanisms described above. Leptin drives ROS formation through other mechanisms validated in different studies [58,59]. In aortic endothelial cells, leptin increases fatty acid oxidation, which results in ROS formation [58]. Leptin induces ROS in both normal (HMECs) and cancerous (MCF-7 and MDA-MB-231) mammary epithelial cells, probably through the activation of NADPH oxidase 5 [59]. 

Another adipokine, resistin, is increased in adipocytes under obesity-associated metabolic conditions, and the expression is upregulated in the breast tissue of diet-induced obese mice [60,61]. In smooth muscle cells, resistin increases intracellular ROS levels through increased activity of the NADPH oxidase [62]. In 2010, Chen et al. found an increase in ROS in coronary artery endothelial cells cultured with resistin for 24 h [63]. 

FABP4 is upregulated in the adipose tissue in obesity [64]. In an in vitro study on pulmonary epithelial cells, FABP4 induced an increase in both ROS levels and pro-inflammatory cytokines (IL-1β, IL-6, and TNF-α) [65]. Hence, FABP4 has the potential to further increase ROS through the increased levels of the cytokines. Furthermore, an increase of ROS levels in bronchial epithelial cells treated with FABP4 was found in another in vitro study [66].

In conclusion, the outlined biomarkers upregulated in the adipose tissue in obesity could induce DNA damage—mainly through ROS production—and thereby, potentially, initiation of cancer (Table 1). Whether similar mechanisms apply to breast tissue remains to be established. Since the majority of the studies are based on other cell types than mammary epithelial cells, further studies are needed to determine if the mechanisms described above constitute the association between the increased breast cancer risk in women with obesity.

## 4. Breast Cancer Progression and Local Obesity-Associated Biomarkers

### 4.1. Inflammatory Biomarkers—Cells and Soluble Factors

Inflammation is a hallmark of cancer, and inflammation in the obese breast is considered a significant link between obesity and breast cancer progression [18,67,68]. As presented above, the adipose tissue in the obese breast is in a chronic state of inflammation, with an altered immune cell landscape and adipose-derived factors, such as pro-inflammatory cytokines. The following will describe local obesity-associated inflammatory biomarkers (cells and soluble factors) in the low-grade inflamed obese breast associated with breast cancer progression (e.g., growth, migration, invasion, and proliferation), Table 2.

#### 4.1.1. Cells

##### Tumor-Associated Macrophages

In obese adipose tissue, the number of macrophages increases compared to lean adipose tissue [71,105]. Traditionally, macrophages are divided into two phenotypes—the pro-inflammatory M1 profile and the anti-inflammatory M2 profile [106]. Under obese conditions, the majority of the increased amount of macrophages skew towards the M1 profile, but an increase in M2-like macrophages is associated with obesity as well according to recent research [107,108,109,110]. Macrophages adjacent to the cancer cells, tumor-associated macrophages (TAMs), have been linked with breast cancer growth and progression [111]. In breast cancer, TAMs can account for more than 50% of the cells within the tumor [69]. Looking at breast cancer prognosis, high density of TAMs is associated with poor disease-free and overall survival [69,70]. Hence, TAMs could serve as a prognostic factor for breast cancer. TAMs play a major role in the progression of tumors, for example, by secretion of different cytokines, chemokines, and proteases [112]. The potential for M1 and M2 macrophages in TAMs in the progression of breast cancer is addressed below.

M1 and M2 macrophages may represent two extremes of the TAMs, according to a review by Qiu et al. [69]. The M1 macrophages secrete pro-inflammatory cytokines, including TNF-α, IL-1β, and IL-6 [71]. These pro-inflammatory cytokines—secreted by, for example, M1 macrophages—are all involved in breast cancer progression, which will be reviewed later in detail (Section 4.1.2).

Even though “M1-like cytokines” are the main source of tumor-promoting inflammatory cytokines in the tumor microenvironment [113], some literature suggests, that TAMs are closely related to the M2 macrophage [69,114]. M2 macrophages are considered “anti-inflammatory” and produce anti-inflammatory cytokines, such as IL-4, IL-10, and IL-13 [114]. The secretion of IL-10 is considered pro-tumorigenic, but also the secretion of matrix metalloproteinases (MMPs), vascular endothelial growth factor A (VEGF-A), chemokine (C-C motif) ligand 18 (CCL-18), programmed death-ligand 1 (PD-L1), and transforming growth factor beta (TGF-β) have shown tumor-promoting features [8,69]. In the obese dysfunctional adipose tissue, M1 macrophages dominate overall, but as mentioned above, various studies have also shown an increase in M2 macrophages. Obesity induces the recruitment of TAMs with an M2-like profile [108]. Further, in a study with 272 breast cancer patients, the number of M2-like TAMs in the tumor was positively correlated with BMI [109]. Springer et al. showed that obesity was associated with an increase in M2-like macrophages in the human breast tissue [110]. In triple-negative breast cancer (TNBC), M2 macrophages seem abundant in the tumor stroma [115].

In conclusion, M1 and M2 macrophages are an abundant part of the TAMs and are increased in obese settings, with most literature classifying TAMs as M2-like. Both the M1 and M2 macrophages could be a part of the link between obesity and inferior prognosis in breast cancer patients. However, there are challenges with this hypothesis. In recent years, research has questioned the classic M1-M2 paradigm, concluding that plasticity and different subpopulations exist [114,116]. Kratz et al. proposed that macrophages in metabolic dysfunction (“obesity”) are activated through two different pathways (toll-like receptors versus p62 and peroxisome proliferator-activated receptor γ) [116]. The balance between these pathways can produce complex macrophage phenotypes spanning the spectrum between M1 and M2 macrophages [116]. Furthermore, a recent review by Tao et al. criticized early research of mistakenly classifying TAMs as M2-like [117]. Supporting this statement, some studies have shown that TAMs are a unique subpopulation of macrophages with both M1- and M2-like features [114]. Further studies are needed to classify the phenotype of TAMs in both patients with and without obesity and to study the potential of TAMs as a prognostic biomarker.

##### Crown-Like Structures

Apart from the TAMs, recent research has also focused on macrophages not closely associated with breast tumors [72]. When the adipose tissue expands, adipocyte hypertrophy occurs in the breast, leading to adipocyte stress and death [8]. Consequently, macrophages are recruited and encircle the adipocytes, forming a crown-like pattern [118]. These patterns are called “crown-like structures” (CLS) and are considered a local biomarker of inflammation [18,119]. The presence of CLS is positively associated with BMI in breast cancer patients [72,120,121,122]. In obese mice, a significant presence of CLS compared to wild-type mice has been reported by Subbaramaiah et al. [123]. Unfortunately, studies investigating the association between BMI and CLS presence in non-breast-cancer patients are sparse. Two smaller case-control studies in patients with benign breast disease (BBD) showed conflicting results, with one reporting a positive correlation between CLS presence and BMI and the other reporting no significant association [124,125]. Furthermore, the positive correlation between CLS and BMI reported by Carter et al. [124] was mainly driven by the patients with BBD, indicating a strong need for studies investigating the association between CLS and BMI in patients without a breast disease. Details regarding the association between breast cancer and CLS have recently been presented in a review by Maliniak et al. [8].

When investigating the association between CLS and breast cancer progression, we focus on studies addressing the role of CLS as a prognostic biomarker. So far, to our knowledge, five studies have examined the impact of CLS and prognosis in breast cancer patients [33,72,73,120,126]. Results have varied, which could be explained by the lack of power due to small sample sizes and varying study methods; for example, in the methods used to detect CLS, since different macrophage markers, such as CD68 and CD163, are used. The majority of the studies have shown a positive association between the presence of CLS and impaired prognosis (defined by impaired disease-free, recurrence-free, progression-free or overall survival). The most recent study, performed by Chang et al. [72], showed a poor distant disease-free survival (adjusted HR: 2.81, 95% CI: 1.20 to 6.57) and overall survival (adjusted HR: 3.97, 95% CI: 1.66 to 9.48) in patients with CLS, detected with the macrophage marker CD68, compared to patients with absent CLS (*n* = 119). The cohort consisted of patients from a single institution in Toronto, Canada with early-stage breast cancer [72]. However, the largest study conducted so far (*n* = 319) reported no association between CLS and progression-free or overall survival [120]. The cohort consisted of both African-American and white women with early-stage breast cancer [120]. As pointed out by Maliniak et al. [8], this study seems methodologically robust, but only one tissue specimen per patient was used in the assessment of the CLS in the breast [120], whereas Iyengar et al. used five breast white adipose tissue sections per patient in their study [33]. That study was conducted in a cohort consisting of 127 patients with early-stage breast cancer, who underwent mastectomy between January 2001 and November 2006 and developed distant metastatic disease within follow-up through 2014 [33]. Thus, all patients ultimately experienced breast cancer progression, which is the outcome of particular interest in this review. CLS were associated with a shortened distant recurrence-free survival (adjusted HR: 1.83, 95% CI: 1.07–3.13) [33]. In the study performed by Koru-Sengul et al. [73] (*n* = 150), CLS were detected using three different macrophage markers; CD206, CD40, and CD163. The authors found a positive association between overall survival and CD40-detected CLS (adjusted HR: 9.14, 90% CI: 1.00 to 83.60) and CD163-detected CLS (adjusted HR: 2.14, 95% CI: 0.46 to 9.96). Contrary to these findings, CD206-detected CLS were associated with a negative association with overall survival (adjusted HR: 0.65, 90% CI: 0.03 to 12.5) [73]. The imprecise measures in that study seem evident, exemplified through the wide confidence intervals. Cha et al. used CD68 and CD163 to detect CLS (*n* = 140), reported that CLS status had no impact on the prognosis of breast cancer upon univariate analysis, but did report that CD68-detected CLS were associated with shorter overall survival in node-positive breast cancer patients [126]. Due to small study populations, different methods, and conflicting results, it is still not evident that CLS could serve as a prognostic biomarker in breast cancer patients.

##### CD8^+^ T cells

As mentioned, the obese adipose tissue enters a pro-inflammatory state with the recruitment of immune cells, for example, CD8^+^ T cells [18,107]. CD8^+^ T cells are essential in the antitumor immune defense, directly killing tumor cells through the release of cytotoxic granules and indirectly promoting tumor rejection by stimulating antigen-presenting cells [74]. High intratumoral CD8^+^ T cell infiltration is associated with improved survival in breast cancer patients [75,76,77]. Thus, an increased number of CD8^+^ T cells, as we see in obese adipose tissue [107], indicates a potential protective mechanism against breast cancer in obesity. On the contrary, obesity-associated mechanisms can alter the function of CD8^+^ T cells and neutralize their anti-tumorigenic potential through multiple mechanisms involving PD-L1 and programmed cell death 1 (PD-1) [127,128,129,130,131]. PD-L1 binds to PD-1 (on, for example, CD8^+^ T cells), consequently impairing CD8^+^ T cell function, leading to tumor progression [18,132]. In breast cancer models, tumor cells in obese settings upregulate the amount of interferon-γ mRNA, a known inducer of PD-L1 on immune cells, for example, myeloid-derived suppressor cells [127]. Furthermore, hypoxia—as seen in obese adipose tissue—upregulates the expression of PD-L1 in macrophages, dendritic cells, and tumor cells through hypoxia-inducible factor 1 alpha [128]. In mice fed with a high-fat diet, the expression of PD-1 in CD8^+^ T cells in white adipose tissue is increased compared to normal mice [129]. In diet-induced obese mouse models of breast cancer, tumor-infiltrating CD8^+^ T cells showed increased expression of PD-1 [130]. Furthermore, mature adipocytes express abundant levels of PD-L1 [131], which could be enhanced in obese settings in the breast due to increased amounts of mature adipocytes. To sum up, an increased amount of CD8^+^ T cells in obesity could protect against breast cancer progression, but increased expression of PD-L1 and PD-1 seem to alter the anti-tumorigenic activities in CD8+ T cells. 

Although breast cancer grows in an adipose-rich environment, breast cancer generally lacks a response to immune checkpoint inhibitors [133]. This could be explained by the above-outlined mechanisms impairing CD8^+^ T cell function in obesity, but also because most breast cancers, except TNBC, are considered “immunologically cold” with a relatively low T-cell infiltration [133]. However, it could be possible that breast cancer patients with obesity could benefit from anti-PD-1/PD-L1 treatment compared to patients without obesity [133]. In melanoma, non-small cell lung cancer, and renal cell cancer, patients with overweight/obesity showed high efficacy of anti-PD-1/PD-L1 treatment compared to normal-weight patients [134]. To our knowledge, this association is yet to be seen in trials on breast cancer patients. In summarization, obesity increases the amount of the anti-tumorigenic CD8+ T cells in the breast, but the potential benefit seems to be neutralized by an increased amount of PD-L1 and PD-1 in the obese adipose tissue. Studies are needed in exploring the possible better anti-PD-1/PD-L1 treatment response in breast cancer patients with obesity versus without obesity. 

#### 4.1.2. Soluble Factors

##### Cytokines

As previously stated, levels of pro-inflammatory cytokines are elevated in the obese breast. In obesity, mature adipocytes increase the secretion of cytokines compared to mature adipocytes in normal adipose tissue [135]. Pro-inflammatory cytokines (IL-1β, IL-6, and TNF-α) not only play a potential role in the initiation of breast cancer (Section 3.2.1), but also in the progression of breast cancer [135,136,137,138]. The proposed mechanisms are outlined below.

##### TNF-α

In both in vitro and in vivo studies, TNF-α has shown potential in breast cancer progression and is considered as one of the most important cytokines in the tumor microenvironment, and is secreted by stromal cells (mainly adipocytes and macrophages) and cancer cells [78,79,80,81,82,83,84,136,137]. In studies on mice, TNF-α increases tumor growth, and blockage of TNF-α through antibodies is correlated with a decrease in tumor size [78,79]. In many breast cancer cell lines, TNF-α contribute to progression in different ways. In MDA-MB-468 (ER-negative) and SK-BR3 (HER2-positive) breast cancer cell lines, TNF-α induces growth [80]. In the ER-positive cell line, T47D, proliferation was induced by TNF-α through several pathways, for example through NF-κB activation [78,81]. TNF-α also promotes migration in the MDA-MB-231 TNBC cell line through upregulation of matrix metalloproteinase 9 (MMP-9) [82]. In adipose tissue, TNF-α stimulates aromatase expression, and thereby indirectly contributes to breast cancer progression (see below) [4,83]. 

Even though TNF-α displays a pro-tumorigenic role, as seen above, contradictory mechanisms have been reported. A review by Cruceriu et al. addresses the contradictory functions of TNF-α in detail [84]. For instance, pro-apoptotic activities were seen in both MCF-7 (ER-positive) and BT-549 (triple-negative) breast cancer cell lines, highlighting the potential dual role of TNF-α in breast cancer progression [84]. Further, no mitogenic action of TNF-α was found in the ER-positive cell line, MCF-7, in a study by Rubio et al. [81]. To sum up, the role of TNF-α in breast cancer progression seems possible, but research is needed to further clarify the contradictory mechanisms by TNF-α.

##### IL-6

Several studies have examined the potential role of IL-6 in breast cancer progression [85,86,87,88,89,90,91]. In the tumor microenvironment, stromal cells and cancer cells act as the major source of IL-6, and the expression of IL-6 is increased in breast tumors [91]. Most studies have focused on the role of IL-6 in invasion, migration, and hereby the metastasis of breast cancer cells. In both ER-positive (MCF-7) and MDA-MB-231 TNBC cell lines, IL-6 promotes invasion and migration of the breast cancer cells [85]. In another in vitro study, IL-6 induced an epithelial-mesenchymal transition (EMT) phenotype in four ER-positive cell lines, through for example, down-regulation of E-cadherin, a membrane adhesion molecule involved in the mobilization of tumor cells [86,91]. Consequently, the migration potential of the breast cancer cells increases through IL-6 treatment. EMT is considered a critical mechanism in cancer progression and is involved in invasion and metastasis [139]. Furthermore, IL-6 promotes breast cancer metastasis through upregulation of lysyl hydroxylase-2, an enzyme, which levels in the tumor correlate with poor prognosis in breast cancer patients [87]. In the study, depletion of PLOD2 (the gene encoding lysyl hydroxylase-2) reduced the MDA-MB-231 TNBC cell migration and invasion [87]. 

Apart from the significant role of IL-6 in invasion, migration, and thus metastasis in breast cancer, IL-6 induces proliferation in MCF-10 ductal carcinoma in situ (DCIS) cell lines, which could contribute to the progression of DCIS to invasive breast cancer [88]. In HER2-positive breast cancer, IL-6 enhances breast cancer progression through expansion of the cancer stem cell population [89]. In addition, IL-6 induces breast cancer cell proliferation indirectly through an increase in estrogen at the tumor site, for example, through activation of the enzyme aromatase [46]. However, the effect of IL-6 on breast cancer cell growth is contradictory, with both inhibitory and promoting effects on proliferation shown in different studies on breast cancer cell lines, as reviewed by Dethlefsen et al. [90].

##### Other Cytokines (IL-1β, IL-8, and IL-10)

IL-1β is abundant in the tumor microenvironment and is secreted by innate immune cells [140]. Obesity leads to an increase in IL-1β production by TAMs [93]. Kolb et al. identified an obesity-induced increase in TAMs with activated NLRC4-inflammasome, which led to an activation of IL-1β [93]. IL-1β, in turn, contributed to tumor progression through upregulation of VEGF-A, hereby promoting angiogenesis [93]. In addition, obesity induces NLRC4/IL-1β-dependent upregulation of angiopoietin-like 4, leading to increased angiogenesis and growth in tumors in mice [94]. Growth of murine 4T1 mammary tumors mediated by IL-1β was also found in another mouse study [95]. IL-1β is also linked to the migration and invasion in breast cancer, for example through loss of E-cadherin and an increase in MMP-2 and MMP-9, leading to a degradation of the extracellular matrix [96,97]. Interestingly, a translational study by Tulotta et al. suggested that IL-1β could be used as a predictive biomarker since they concluded that the production of IL-1β by the breast cancer cells promoted bone metastasis [98]. Another pro-inflammatory cytokine upregulated in obesity, IL-8, is associated with breast cancer proliferation and invasion [99,100,136]. For instance, IL-8 secreted by mammary adipocytes increases the dissemination capacity of breast cancer cells [99], and IL-8 enhances the tumorigenesis-promoting effects of adipocytes closely related to the tumor, the so-called cancer-associated adipocytes (CAAs) [100]. CAAs release a major amount of the biomarkers addressed in this review, but the role of cancer-associated adipocytes in detail will not be reviewed here. Recent reviews have covered the current knowledge on CAAs very well [141,142,143]. In a study on mice, the levels of the anti-inflammatory cytokine, IL-10, were reduced in the mammary fat pad with increased adiposity [83]. Furthermore, the authors concluded that IL-10 suppresses aromatase expression in human breast adipose stromal cells and thereby moderates the aromatase-induced breast cancer progression. However, IL-10 also induces pro-tumorigenic effects [69], exemplified in another study on mice by Kaplanov et al. [95]. In that study, IL-10 secretion from macrophages induced tumor progression through CD8^+^ T cell suppression, pointing towards an opposite role of IL-10 in breast cancer progression [95]. 

To summarize, pro-inflammatory cytokines in the obese breast seem to take part in the association between obesity and breast cancer progression, but the association remains incompletely mapped. So far, studies point towards a pro-tumorigenic role for most of the obesity-associated cytokines, whereof some, i.e., TNF-α, IL-6, and IL-10, also seem to induce activities inhibiting tumor progression.

##### Chemokines

In the obese adipose tissue, the secretion of chemokines [chemokine (C-C motif) ligand 2 (CCL-2) and 5 (CCL-5)] increases compared with non-obese adipose tissue [135,144]. CCL-2 [also called monocyte chemoattractant protein-1 (MCP-1)] acts as a chemoattractant and recruits immune cells, for example monocytes/macrophages [145]. Stromal CCL-2 in breast tumors correlates with infiltrations of TAMs, which contributes to breast cancer progression as earlier described [146]. A high expression of CCL-2 in breast cancer tissue is found to be a significant indicator of early relapse [147], metastasis [148], and upregulation of CCL-2 in breast cancer tissue reduces overall survival [149]. Overexpression of CCL-2 induces cell invasion and metastasis in TNBC [101]. 

Another chemokine, CCL-5, shows similar abilities as CCL-2 in breast cancer progression, for example, through the attraction of TAMs [102]. CCL-5 released from adipocytes promotes motility and invasiveness in MDA-MB-231 TNBC cell lines [103]. In the same translational study, the abundance of CCL-5 in peritumoral adipose tissue correlated with lymph node status and metastasis [103]. Song et al. discovered that increased secretion of CCL-5 by adipocytes enhanced the EMT effect of MDA-MB-231 and MDA-MB-453 TNBC cell lines, thereby promoting tumor growth and metastasis [104]. A significant difference in CCL-5 expression in breast tumors between stage I and stage III patients was discovered by Derossi et al. [150], suggesting a possible role for CCL-5 in the aggressiveness of the tumor. In addition, overexpression of CCL-5 in HER2-positive breast cancer is associated with poor disease-free survival and lower overall survival [151]. It is evident that CCL-2 and CCL-5 could act as prognostic local biomarkers and are involved in breast cancer progression, but further research is needed since many of the included studies have small sample sizes.

### 4.2. Aromatase Expression and Estrogens

As mentioned, the local level of aromatase-expression, and thereby 17β-estradiol (E2), is increased in obese adipose tissue. Estrogens not only contribute to the initiation of breast cancer (Section 3.2.2) but are also involved in breast cancer progression. More than 75% of breast tumors express the estrogen receptor (ER), and a review by Gérard et al. stated that current knowledge suggests that the association between obesity and postmenopausal breast cancer is highest among the hormone-receptor-positive cases [92]. 

Activation of the ER drives a variety of cell functions involved in progression, such as growth, angiogenesis, and migration [92]. E2 binds to its receptor, ER, translocates to the nucleus (“genomic” actions), and binds to estrogen response elements (ERE) on genes promoting cancer growth by increasing proliferation and inhibiting apoptosis [152]. The regulation of ERE is important in many pathological processes, for example, tumor progression and carcinogenesis [92]. In 2019, Morgan et al. discovered that the transactivation of ERE was heightened in the ER-positive MCF-7 breast cancer cell lines co-cultured with adipose stromal cells from individuals with obesity due to increased aromatase expression, further showing the amplified effect of estrogens on breast cancer progression in obesity [153]. In addition, estrogens can act beyond the ERE, both in ERE-independent genomic activation and through non-genomic (“extra-nuclear”) pathways [92]. For instance, estrogens contributed to the migration and invasion of breast cancer cells in an in vitro study through an extra-nuclear pathway involving G protein Gα_13_ [154]. Further details on both the genomic (ERE-dependent and non-dependent) and non-genomic effects of estrogens have recently been outlined in other reviews [48,92] and will not be further addressed here. Looking beyond ER-positive breast cancer, E2 potentiates the growth of 4T1 mouse ER-negative breast tumor cell lines via increased angiogenesis, suggesting a potential role in estrogen-negative tumors [155]. In vitro, E2 also promoted the invasion and migration of ER-negative breast cancer cell lines [156]. In conclusion, estrogen in the local environment is suspected to be a key driver of breast cancer progression, with the effect further enhanced in individuals with obesity.

### 4.3. Adipokines

Adipokines can affect adjacent breast cancer cells in the tumor microenvironment, which has been suggested to play a critical role in breast cancer progression [53,157]. Leptin and adiponectin are the most studied adipokines in breast cancer progression, but other adipokines were discovered contributing to breast cancer progression as well [53]. In the following section, the role of dysregulated levels of adipokines in obesity in breast cancer progression will be discussed.

#### 4.3.1. Leptin

As previously stated, local levels of leptin are increased in obese adipose tissue and are believed to strongly contribute to breast cancer progression [54]. Leptin acts through its receptor, Ob-R, leading to signal transduction through phosphorylation of JAK2 and downstream activation of several pathways, for example, STAT3 and PI3K/AKT, resulting in the transcription of genes involved in proliferation, angiogenesis, invasion, migration, and cell survival [3,157]. A study by Ishikawa et al. found overexpression of Ob-R in invasive ductal breast cancer as compared to normal mammary epithelial cells [158]. A combination of higher levels of leptin under obese conditions and increased expression of Ob-R in breast cancer cells further suggests a vital role of leptin in obesity-associated breast cancer. 

In various studies, both in vivo and in vitro, the role of leptin in breast cancer progression has been addressed [53,159,160,161,162,163,164,165,166,167,168]. In MCF-7 (ER-positive) breast cancer cells, leptin increased proliferation through a STAT3-dependent pathway [159,160]. In addition, leptin inhibits apoptosis by downregulating pro-apoptotic genes in MCF-7 (ER-positive) breast cancer cells [161]. In MCF-7 (ER-positive) and MDA-MB-231 TNBC cells, leptin induces invasive potential through increased secretion of metalloproteinases (MMP-2 and MMP-9) [168]. In TNBC and ER-positive breast cancer cell lines, leptin from obese adipose stromal cells promotes metastasis through upregulation of EMT and other metastasis genes [162,163]. The EMT process and promotion of breast cancer are further suggested to be stimulated by progenitor cells in white adipose tissue, represented by both endothelial cells and adipose stromal cells [169]. The role of adipose progenitor cells in obesity has been thoroughly addressed in a review by Reggiani et al. [19]. In a 4T1 mouse mammary cancer model, leptin increased the expression of VEGF, thereby promoting angiogenesis leading to breast cancer progression [164]. Leptin also acts by enhancing the effect of previously outlined obesity-associated biomarkers. Leptin enhances the aromatase expression in the MCF-7 (ER-positive) cell line and can activate the ER in the same cell line, implying that leptin can induce breast cancer progression through the previously outlined effects by estrogens [165,166]. Furthermore, leptin mediates the production of the above addressed pro-inflammatory cytokines involved in breast cancer progression; IL-1β, IL-6, and TNF-α [167]. CD8^+^ T cell dysfunction is also enhanced by leptin through upregulation of PD-1, thereby opposing the antitumor function by these immune cells [130].

#### 4.3.2. Adiponectin

Adiponectin has also been linked with breast cancer progression but has shown mostly anti-tumorigenic effects [170,171,172,173,174]. The effect on breast cancer progression seems to depend on ER status [170]. With increasing BMI, the levels of adiponectin in serum decreases [175]. Unfortunately, to our knowledge, the regulation of the adiponectin levels in the local adipose tissue or adipocytes in obese versus non-obese states has not been investigated [135]. In many in vitro and in vivo studies investigating the role of adiponectin in ER-negative cell lines, adiponectin inhibits breast cancer progression [171,172,173]. In MDA-MB-231 TNBC cell lines, adiponectin suppresses proliferation, induces apoptosis, and inhibits invasion [171,172]. Moreover, adiponectin reduces the mammary tumorigenesis of MDA-MB-231 TNBC cells in mice, as found by Wang et al. [174]. With regards to ER-positive tumors, the results are conflicting. Studies on MCF-7 (ER-positive) cell lines have shown both anti-tumorigenic, pro-tumorigenic, and neutral effects on progression by adiponectin [170,176,177,178,179,180,181,182]. Adiponectin can induce anti-progression mechanisms in MCF-7 (ER-positive) cell lines, for example, through increased cell apoptosis and decreased cell proliferation [176,177]. However, in both in vivo (xenografted mouse study) and in vitro studies (on ER-positive MCF-7 cells), Mauro et al. found that a low level of adiponectin (5 μg/mL), corresponding to the plasma level in women with obesity, increased cell and tumor growth, and a higher level of adiponectin (30 μg/mL), corresponding to the plasma level in normal-weight women, had no significant effect on cell and tumor growth [178,179,180]. Through activation of the ER in both genomic and non-genomic ways, adiponectin can induce breast cancer progression in ER-positive breast cancer [181]. For example, it is suggested that adiponectin at low levels upregulates the expression of cyclin D1 through recruitment of the ER to its promotor, and hereby induces cell proliferation [178,179,181]. However, adiponectin does have an inhibitory effect on the ER activity through inhibition of aromatase expression in breast adipose tissue [182]. In conclusion, the effect of adiponectin differs according to ER status, and further investigation is needed. Studies show a trend towards anti-proliferative effects in ER-negative tumors, and the effects in ER-positive cells and tumors are more diverse and differ.

#### 4.3.3. Resistin

In 2001, resistin, for ”insulin resistance”, was labeled as a potential mediator between obesity and diabetes [183]. Resistin has been linked with breast cancer progression and could contribute to the link between obesity and breast cancer, as previously reviewed [53,135,184]. Resistin is secreted from adipocytes and immune cells (e.g., macrophages) [185]. As mentioned previously, the levels of resistin are increased in obesity [60,61]. Resistin has been linked with several mechanisms resulting in breast cancer progression [186,187,188,189], and upregulation of resistin in breast cancer tumors is associated with impaired prognosis [190,191]. In MDA-MB-231 TNBC cell lines, resistin enhances invasion and migration of the breast cancer cells, thereby promoting metastasis [186]. In a study on MCF-7 (ER-positive) and MDA-MB-231 TNBC cell lines, further potential regarding metastasis was shown by Avtanski et al., as resistin upregulated the expression of EMT-markers, such as SNAIL, TWIST1, and fibronectin, and downregulated the previously addressed epithelial marker E-cadherin [187]. In the same cell lines, resistin promotes EMT and stemness, and hereby breast cancer progression, through activation of toll-like receptor 4 signaling [188]. Resistin also induces growth in MDA-MB-231 TNBC and MDA-MB-468 (ER-negative) cell lines, according to Deshmukh et al. [189]. In 2018, Rosendahl et al. found that the resistin-receptor, adenylyl cyclase-associated protein-1 (CAP1), was expressed across different breast cancer subtypes, with higher expression found in ER-negative tumors compared to ER-positive tumors [60]. In addition, high CAP1 expression was associated with poor breast cancer outcomes in all subtypes [60]. In a study on T47D (ER-positive) and MDA-MB-231 TNBC cell lines, silencing of CAP1 decreased cell proliferation [192]. To sum up, resistin in the tumor microenvironment could promote breast cancer progression, for example, through activation of the CAP1. 

#### 4.3.4. Other Adipokines

The adipokines plasminogen activator inhibitor 1 (PAI-1), FABP4, and secreted frizzled-related protein 5 (SFRP5) are also found in the local environment of the breast and have been shown to contribute to breast cancer progression [64,193,194,195,196,197,198,199]. Higher levels of the adipokine, PAI-1, in breast cancer were found to correlate with poor prognosis in breast cancer patients [193]. PAI-1 is mostly secreted from visceral adipose tissue and increased in obese states [194,195], but the preadipocytes and adipocytes in the breast express PAI-1 as well [196]. PAI-1 has been suggested to be involved in different mechanisms in breast cancer progression, including angiogenesis and migration, as reviewed by Carter et al. [195]. In individuals with obesity, the adipokine FABP4 is upregulated in the adipose tissue and is linked to obesity-related breast cancer [64]. Focusing on the mechanisms in breast cancer progression, FABP4 enhances proliferation in both MCF-7 cells (ER-positive) and MDA-MB-231 TNBC cells but does not appear to affect the migration potential [197]. FABP4 is involved in the lipid metabolism in the tumor microenvironment, and suppression of FABP4 reduces the lipid transfer between adipocytes and cancer cells, which could explain the observed proliferative response in the above-mentioned study [198]. Secreted SFRP5 is a fairly novel adipokine in the research area regarding the association between obesity and breast cancer, as reviewed by Zhao et al. [53]. In that review, a study published in 2020 by Zhou et al. is addressed [199], demonstrating decreased circulating levels of SFRP5 in patients with obesity, and that high levels of SFRP5 in the tumor tissue were associated with better outcomes [199]. In a hypertrophic adipocyte model, mimicking obese states, cell migration and invasion of MCF-7 (ER-positive) and MDA-MB-231 TNBC cells were promoted, but these effects were reduced when adding SFRP5 [199]. SFRP5 inhibited EMT pathways in the breast cancer cell lines, thereby suppressing the invasive potential and, therefore, breast cancer progression [199]. 

In summary, various adipokines in obesity seem to participate in breast cancer progression (Table 3). On the one hand, leptin and resistin, among others, display pro-tumorigenic properties. On the other hand, adipokines with lower levels in obesity, such as adiponectin and SFRP5, act as anti-tumorigenic agents, and the loss of these effects promotes tumor progression.

### 4.4. Extracellular Matrix Remodeling

The non-cellular part of the tumor microenvironment is called the extracellular matrix (ECM). The remodeling of ECM, a process called desmoplasia, is regulated by myofibroblasts (“activated fibroblasts”), which create an ECM rich in fibronectin and collagens, essentially leading to a fibrotic and stiff ECM [18,24,200,201]. The tumor tissue is stiffer than healthy tissue, and an association between the level of ECM stiffness and breast cancer aggression has been found [202]. The number of myofibroblasts and fibronectin in mammary adipose tissue in mice increases during obesity, resulting in ECM stiffness [24]. Further, macrophages in CLS, which are upregulated in the obese adipose tissue, promote myofibroblast activation and ECM stiffness [18]. Consequently, remodeling of the ECM is believed to contribute to breast cancer progression in obesity [24,143]. In 2015, Seo et al. found, that decellularized ECM deposited by obesity-associated adipose stem cells (in which a larger part consists of myofibroblasts in obesity) stimulated the mechanosensitive growth of the MDA-MB-231 TNBC cell line [24]. ECM remodeling seems to enhance breast cancer progression, and ECM remodeling and fibrosis appear distinct in obese adipose tissue [203]. It is evident, that myofibroblasts play a major role in the ECM remodeling and breast cancer progression in obesity. In the following section, we will address some of the proposed biomarkers in the altered ECM in obesity involved in breast cancer progression apart from the myofibroblasts in general (Table 4).

#### 4.4.1. Matrix Metalloproteinases (MMP-9 and MMP-2)

Myofibroblasts and adipocytes are major sources of matrix metalloproteinases, for example, MMP-2 and MMP-9, two MMPs receiving attention in research focusing on breast cancer progression [14,212,213,214]. Matrix metalloproteinases can promote cancer cell invasion by disrupting cell-cell adhesion, for example, through cleavage of E-cadherin [204,205]. MMP-9 plays a role in angiogenesis, growth, and metastasis in breast cancer, consequently resulting in breast cancer progression, with MMP-9 being a potential negative prognostic marker [82,206,207]. Ramos-Andrade et al. [215] found that extracellular vesicles released from obese adipose tissue were enriched in MMP-9, but others have found no difference in MMP-9 levels in obese versus lean tissue in mice [216]. Lower levels have also been found in gonadal adipose tissue in obese mice [217]. However, previously described biomarkers linked to obesity, such as TNF-α and IL-1β, upregulate the levels of MMP-9, suggesting a potential link between obesity, MMP-9, and breast cancer progression [82,97]. MMP-2 mRNA levels in adipose tissue correlate positively with obesity [216]. In MCF-7 (ER-positive) breast cancer cell lines, upregulation of MMP-2 significantly correlated with invasiveness, and metastatic human breast cancer tumors showed higher levels of MMP-2 than non-metastatic tumor tissue [208]. 

#### 4.4.2. Collagen VI and Endotrophin

In the obese adipose tissue, the amount of collagens, including collagen VI, is upregulated [18]. Collagen VI promotes the growth of breast cancer cells through the NG2/chondroitin sulfate proteoglycan receptor [209]. However, a major part of the role of collagen VI has been suggested as the result of a cleavage product called endotrophin. The level of endotrophin is upregulated in the adipose tissue of obese mice, compared to lean mice, and the circulating levels of endotrophin are elevated in breast cancer patients compared to non-breast-cancer patients [210,211]. Endotrophin leads to EMT via TGF-β signaling and therefore, contributes to breast cancer progression [210]. Additionally, EMT-markers are upregulated in T47D (ER-positive) cell lines that are treated with endotrophin [211]. These findings were replicated in MCF-7 (ER-positive) and MDA-MB-231 TNBC cell lines in the same study, indicating a potential role for endotrophin in various breast cancer subtypes [211].

In conclusion, ECM remodeling is upregulated in obesity and seems to be involved in the progression of breast cancer, and myofibroblasts, MMPs, and collagens seem to be a part of the explanation.

## 5. Conclusion and Future Perspectives

As breast cancer is the most common form of cancer in women worldwide (excluding non-melanoma skin cancer) and the incidence of obesity is increasing, the need for awareness of the association between obesity and breast cancer development and progression is evident. In this review, we have outlined the current landscape on a variety of local obesity-associated biomarkers linked to breast cancer initiation and progression. Inflammatory biomarkers, such as macrophages, cytokines, and chemokines; all seem to participate in the progression of breast cancer and for some, also in the initiation of breast cancer. The amount of active estrogen is upregulated in women with obesity and enhances breast cancer initiation and progression. Further, soluble factors secreted by adipocytes, named adipokines, make for a major contribution to obesity-associated breast cancer. In particular, an increased amount of leptin and decreased amount of adiponectin has been explored widely and seem to be a part of the explanation. Lastly, ECM remodeling and fibrosis in the obese breast creates a pro-tumorigenic environment for the breast cancer cells. So far, many in vitro and in vivo studies have explored the effects of the addressed obesity-associated biomarkers in the breast. However, more studies are needed. The literature describing the mechanisms in the obese breast and breast cancer initiation is sparse, and most included studies in this review are based on other cell types than mammary epithelial cells. Furthermore, in vitro studies mimicking obese settings, investigating the levels of a given biomarker and the subsequent effect on breast cancer cells, have the potential to create a wider understanding of the mechanisms between obesity and breast cancer. More studies on human tissue investigating the difference between obese and non-obese breast tissue in breast cancer patients and non-breast-cancer patients could bring new knowledge in biomarkers involved in the association between obesity and breast cancer initiation and progression. A pilot study published in 2013 quantified the breast tissue levels of different adipokines and cytokines in women with a high risk of breast cancer (*n* = 26) [218,219]. In this relatively small cohort, only leptin significantly correlated with BMI, further demonstrating the gap within the area. Through future research, we should aim to identify women with obesity with an increased breast cancer risk and/or an impaired prognosis through the different levels of the biomarkers in the breast.

## Figures and Tables

**Table 1:** Local obesity-associated biomarkers possibly involved in the initiation of breast cancer.

Biomarker	Level in Obesity Compared to Non-Obesity	Association with Breast Cancer Initiation	References
TNF-α	Increased	TNF-α increases ROS production in myocardial cells and liver cells.	[37,38]
IL-1β	Increased	IL-1β induces ROS production in chondrocytes.	[39,40,41]
IL-6	Increased	IL-6 increases the intracellular production of ROS in normal 3T3-L1 adipocytes.	[42]
Estrogens	Increased	Estrogen metabolism forms catechol estrogen metabolites inducing the production of ROS through redox cycling. These catechol estrogen metabolites can interact directly with the DNA, resulting in point mutations. Estrogens can impair the DNA damage response.	[51,52]
Leptin	Increased	Leptin upregulates the expression of TNF-α, IL-1β, IL-6, and estrogens. Leptin increases fatty acid oxidation in aortic endothelial cells, resulting in ROS formation. Leptin induces ROS in normal and cancerous mammary epithelial cells.	[25,49,56,57,58,59]
Resistin	Increased	Resistin increases ROS levels in smooth muscle cells and coronary artery endothelial cells.	[62,63]
FABP4	Increased	FABP4 induces an increase in both ROS levels and pro-inflammatory cytokines in pulmonary epithelial cells. FABP4 increases ROS levels in bronchial epithelial cells.	[65,66]

Abbreviations: TNF-α = tumor necrosis factor alpha; ROS = reactive oxygen species; IL = interleukin; FABP4 = fatty acid-binding protein 4.

**Table 2:** Local obesity-associated inflammatory biomarkers involved in breast cancer progression.

Biomarker	Level in Obesity Compared to Non-Obesity	Association with Breast Cancer Progression	References
TAMs	Increased	A high density of TAMs is associated with poor disease-free and overall survival. The M1 macrophages secrete pro-inflammatory cytokines, including TNF-α, IL-1β, and IL-6, which are all involved in breast cancer progression. The M2 macrophages secrete pro-tumorigenic factors, such as IL-10, MMPs, VEGF-A, CCL-18, PD-L1, and TGF-β.	[8,69,70,71]
CLS	Increased	The presence of CLS is associated with an impaired prognosis in breast cancer patients.	[33,72,73]
CD8^+^ T cells	Increased	CD8^+^ T cells are essential in the anti-tumor immune defense, for example, through the release of cytotoxic granules, killing tumor cells. High intratumoral CD8^+^ T cell infiltration is associated with improved survival in breast cancer patients.	[74,75,76,77]
TNF-α	Increased	TNF-α increases tumor growth, and blockage of TNF-α through antibodies is correlated with a decrease in tumor size. TNF-α induces growth in MDA-MB-468 (ER-negative) and SK-BR3 (HER2-positive) breast cancer cell lines. TNF-α induces proliferation through several pathways, for example through NF-κB activation, in the ER-positive cell line, T47D. TNF-α promotes migration in the MDA-MB-231 TNBC cell lines through upregulation of MMP-9. TNF-α stimulates aromatase expression in adipose tissue. TNF-α induces pro-apoptotic activities in both MCF-7 (ER-positive) and BT-549 (triple-negative) breast cancer cell lines.	[4,78,79,80,81,82,83,84]
IL-6	Increased	In both ER-positive (MCF-7) and MDA-MB-231 TNBC cell lines, IL-6 promotes invasion and migration. IL-6 induces an EMT phenotype in ER-positive cell lines. IL-6 promotes breast cancer metastasis through the upregulation of lysyl hydroxylase-2. IL-6 induces proliferation in MCF-10 DCIS cell lines. IL-6 enhances breast cancer progression through expansion of the cancer stem cell population in HER2-positive breast cancer. IL-6 induces both inhibitory and promoting effects on proliferation in breast cancer cell lines. IL-6 induces breast cancer cell proliferation indirectly through activation of the enzyme aromatase.	[46,85,86,87,88,89,90,91,92]
IL-1β	Increased	IL-1β contributes to tumor progression through upregulation of VEGF-A, thereby promoting angiogenesis. IL-1β contributes to the upregulation of angiopoietin-like 4, leading to increased angiogenesis and growth in tumors in mice. IL-1β mediates growth in murine 4T1 mammary tumors. IL-1β promotes migration and invasion in breast cancer, for example through loss of E-cadherin and an increase in MMP-2 and MMP-9, leading to a degradation of the extracellular matrix. Production of IL-1β by breast cancer cells promotes bone metastasis.	[93,94,95,96,97,98]
IL-8	Increased	IL-8 secreted by mammary adipocytes increases the dissemination capacity of breast cancer cells. IL-8 enhances the tumorigenesis-promoting effects of CAAs.	[99,100]
IL-10	Decreased	IL-10 suppresses aromatase expression in human breast adipose stromal cells. IL-10 secretion from macrophages induces tumor progression through CD8^+^ T cell suppression.	[83,95]
CCL-2	Increased	Overexpression of CCL-2 induces cell invasion and metastasis in TNBC. CCL-2 attracts TAMs.	[101,102]
CCL-5	Increased	CCL-5 attracts TAMs. CCL-5 released from adipocytes promotes motility and invasiveness in MDA-MB-231 TNBC cell lines. Increased secretion of CCL-5 by adipocytes enhanced the EMT effect of MDA-MB-231 and MDA-MB-453 TNBC cell lines.	[102,103,104]

Abbreviations: TAMs = tumor-associated macrophages; MMP = matrix metalloproteinase; VEGF-A = vascular endothelial growth factor A; CCL = chemokine (C-C motif) ligand; PD-L1 = programmed death-ligand 1; TGF-β = transforming growth factor beta; CLS = crown-like structures; TNF-α = tumor necrosis factor alpha; IL = interleukin; EMT = epithelial-mesenchymal transition; DCIS = ductal carcinoma in situ; CAAs = cancer-associated adipocytes; ER = estrogen receptor; TNBC = triple-negative breast cancer.

**Table 3:** Local obesity-associated adipokines involved in breast cancer progression.

Biomarker	Level in Obesity Compared to Non-Obesity	Association with Breast Cancer Progression	References
Leptin	Increased	In MCF-7 (ER-positive) breast cancer cells, leptin increases proliferation through a STAT3-dependent pathway. Leptin inhibits apoptosis by downregulating pro-apoptotic genes in MCF-7 (ER-positive) breast cancer cells. In MCF-7 (ER-positive) and MDA-MB-231 TNBC cells, leptin induces invasive potential through increased secretion of MMP-2 and MMP-9. Leptin, from obese adipose stromal cells, promotes metastasis through upregulation of EMT and other metastasis genes in TNBC and ER-positive breast cancer cell lines. In a 4T1 mouse mammary cancer model, leptin increased the expression of VEGF, thereby promoting angiogenesis leading to breast cancer progression. Leptin enhances the aromatase expression in the MCF-7 (ER-positive) cell line and can activate the ER in the same cell line. Leptin mediates the production of pro-inflammatory cytokines involved in breast cancer progression; IL-1β, IL-6, and TNF-α. CD8^+^ T cell dysfunction is enhanced by leptin through upregulation of PD-1.	[130,159,160,161,162,163,164,165,166,167,168]
Adiponectin	Decreased	In MDA-MB-231 TNBC cell lines, adiponectin suppresses proliferation, induces apoptosis, and inhibits invasion. Adiponectin reduces mammary tumorigenesis of MDA-MB-231 TNBC cells in mice. Adiponectin can induce anti-progression mechanisms in MCF-7 (ER-positive) cell lines, for example, through increased cell apoptosis and decreased cell proliferation. A low level of adiponectin (5 µg/mL), increases cell and tumor growth, and a higher level of adiponectin (30 µg/mL), has no significant effect on cell and tumor growth. Through activation of the ER in both genomic and non-genomic ways, adiponectin can induce breast cancer progression in ER-positive breast cancer. Adiponectin (at low levels) upregulates the expression of cyclin D1 through recruitment of the ER to its promotor and hereby induces cell proliferation. Adiponectin has an inhibitory effect on the ER activity through the inhibition of aromatase expression in breast adipose tissue.	[171,172,174,176,177,178,179,180,181,182]
Resistin	Increased	Resistin enhances the invasion and migration of MDA-MB-231 TNBC cell lines. In MCF-7 (ER-positive) and MDA-MB-231 TNBC cell lines, resistin upregulates the expression of EMT-markers, such as SNAIL, TWIST1, and fibronectin, and downregulates E-cadherin. In MCF-7 (ER-positive) and MDA-MB-231 TNBC cell lines, resistin promotes EMT and stemness, and hereby breast cancer progression, through activation of toll-like receptor 4 signaling. Resistin induces growth in MDA-MB-231 TNBC and MDA-MB-468 (ER-negative) cell lines. Silencing of CAP1 (resistin-receptor) decreases cell proliferation in T47D (ER-positive) and MDA-MB-231 TNBC cell lines.	[186,187,188,189,192]
PAI-1	Increased	PAI-1 is involved in angiogenesis and migration in breast cancer.	[195]
FABP4	Increased	FABP4 enhances proliferation in both MCF-7 cells (ER-positive) and MDA-MB-231 TNBC cells. Suppression of FABP4 reduces the lipid transfer between adipocytes and cancer cells.	[197,198]
SFRP5	Decreased	SFRP5 reduces cell migration and invasion of MCF-7 (ER-positive) and MDA-MB-231 TNBC cells. SFRP5 inhibited EMT pathways in MCF-7 (ER-positive) and MDA-MB-231 TNBC cells. A high level of SFRP5 in the tumor tissue is associated with better outcomes.	[199]

Abbreviations: ER = estrogen receptor; MMP = matrix metalloproteinase; TNBC = triple-negative breast cancer; VEGF = vascular endothelial growth factor; EMT = epithelial-mesenchymal transition; TNF-α = tumor necrosis factor alpha; IL = interleukin; PD-1 = programmed cell death 1; CAP1 = adenylyl cyclase-associated protein-1; PAI-1 = plasminogen activator inhibitor 1; FABP4 = fatty acid-binding protein 4; SFRP5 = secreted frizzled-related protein 5.

**Table 4:** Local obesity-associated biomarkers in ECM remodeling involved in breast cancer progression.

Biomarker	Level in Obesity Compared to Non-Obesity	Association with Breast Cancer Progression	References
Matrix metalloproteinases	Increased	Matrix metalloproteinases can promote cancer cell invasion by disrupting cell-cell adhesion, for example, through cleavage of E-cadherin. MMP-9 plays a role in angiogenesis, growth, and metastasis in breast cancer, consequently resulting in breast cancer progression. In MCF-7 (ER-positive) breast cancer cell lines, upregulation of MMP-2 significantly correlates with invasiveness.	[82,204,205,206,207,208]
Collagen VI/endotrophin	Increased	Collagen VI promotes the growth of breast cancer cells through the NG2/chondroitin sulfate proteoglycan receptor. Endotrophin leads to EMT via TGF-β signaling. EMT-markers are upregulated when T47D (ER-positive), MCF-7 (ER-positive), and MDA-MB-231 TNBC cell lines are treated with endotrophin.	[209,210,211]

Abbreviations: MMP = matrix metalloproteinase; ER = estrogen receptor; EMT = epithelial-mesenchymal transition; TGF-β = transforming growth factor beta; TNBC = triple-negative breast cancer.
